# The gender-specific bidirectional relations between chronic diseases and total bilirubin/urea in the elderly population: A 3-year longitudinal study

**DOI:** 10.3389/fpubh.2022.1003505

**Published:** 2022-11-09

**Authors:** Na Wu, Xiangyu Zhai, Mofan Feng, Jie Li, Ning Yu, Fengwei Zhang, Dong Li, Jianying Wang, Lei Zhang, Yi Shi, Guang He, Guang Ji, Baocheng Liu

**Affiliations:** ^1^Shanghai Innovation Center of Traditional Chinese Medicine Health Service, Shanghai University of Traditional Chinese Medicine, Shanghai, China; ^2^Key Laboratory for the Genetics of Developmental and Neuropsychiatric Disorders, Bio-X Institutes, Shanghai Jiao Tong University, Shanghai, China; ^3^Graduate School of Sport Sciences, Waseda University, Saitama, Japan; ^4^Zhangjiang Community Health Service Center of Pudong New District, Shanghai, China; ^5^Longhua Hospital, Institute of Digestive Diseases, Shanghai University of Traditional Chinese Medicine, Shanghai, China

**Keywords:** aging, gender-specific, chronic diseases, longitudinal study, bidirectional relation

## Abstract

Aging is accompanied by changes in physiology over time, which remains the largest risk of chronic diseases. The aim of this study was to explore the gender-specific bidirectional relations between the risk of chronic diseases and serum traits in a 3-year longitudinal study. A hierarchical non-linear model with random effects was used to assess the temporal patterns of anthropometric and serum traits from 2017 to 2019 among 2,338 participants. To assess the directional effect between the risk of chronic diseases and serum traits, a bivariate cross-lagged panel model (CLPM) was used to estimate the structural relations of repeatedly measured variables at three different time points. Candidate SNPs were analyzed and genotyped in MassARRAY Analyzer 4 platforms. In this study, metabolic syndrome (MS) score increased with aging in females, whereas the fatty liver disease (FLD) index decreased with aging in males; the MS score was negatively correlated with TB in females, and FLD index was positively related to urea in males; CLPM showed that the MS score predicted total bilirubin (TB) in females, and urea predicted the FLD index in males. Additionally, rs2292354 in G protein-coupled receptor kinase interactor 2 (*GIT2*) was associated with the MS score and TB in aged females. Our study suggests the potential gender-specific causal associations between development in MS and increase in TB level in females, and rise in urea level and improved FLD index in males. The SNP rs2292354 we investigated might be a biomarker for predicting MS in the elderly Chinese Han population.

## Introduction

By the year 2050, the population aged over 60 years old is estimated to increase by nearly 0.4 billion in China (1). Aging is accompanied by changes in physiology over time, which remains the largest risk of chronic diseases, such as neurodegenerative diseases, cardiovascular diseases, metabolic syndrome (MS) and non-alcoholic fatty liver (NAFLD) (2). As major public health problems, these chronic diseases have led to a cumulative burden on society. Thus, it is urgently required to explore the key mechanisms of aging and age-related chronic diseases.

Age-related impairment of endocrine function results from phenotypic alterations of different cell types, such as endothelial cells, cytokine, adipocytes and hepatocytes (3). The mechanism of MS during aging is likely driven by those phenotypic changes. Evidence has shown that bilirubin can function as an antioxidant by reducing reactive oxygen species and suppressing the oxidative activity of nicotinamide adenine dinucleotide phosphate, resulting in oxidative stress alleviation (4), which is involved in the pathogenesis and development of MS (5). In recent cross-sectional studies, Hwang and Kim observed an inverse relationship between total bilirubin (TB) and MS in Korean women (6); Li et al. (7) confirmed the negative association between bilirubin and MS incidence among Chinese men; Zhong et al. (8) found that TB was negatively associated with MS among the aged Chinese women. Although the association of TB with MS appears to be reported a lot, it still lacks the support from longitudinal studies, which might be helpful to clarify the inconsistency between females and males, and further provide the causality clues. Notably, the urea cycle plays an essential role in NAFLD progression (9). The conversion process from nitrogen into urea has been disrupted, especially in the elderly population with chronic diseases; the bidirectional relation between fatty liver and urea is seldom mentioned.

As we all know, genetic factors play essential roles in the occurrence and progression of chronic diseases. While the genetic susceptibility in the aging population with chronic diseases was seldom mentioned.

Our objective was to evaluate whether TB and urea levels change with aging and predict the later development of the MS and NAFLD in the aged females and males by using longitudinal studies. In addition, candidate genes related to chronic diseases in aging population was also investigated.

## Methods and materials

### Subjects

**Figure 1:**
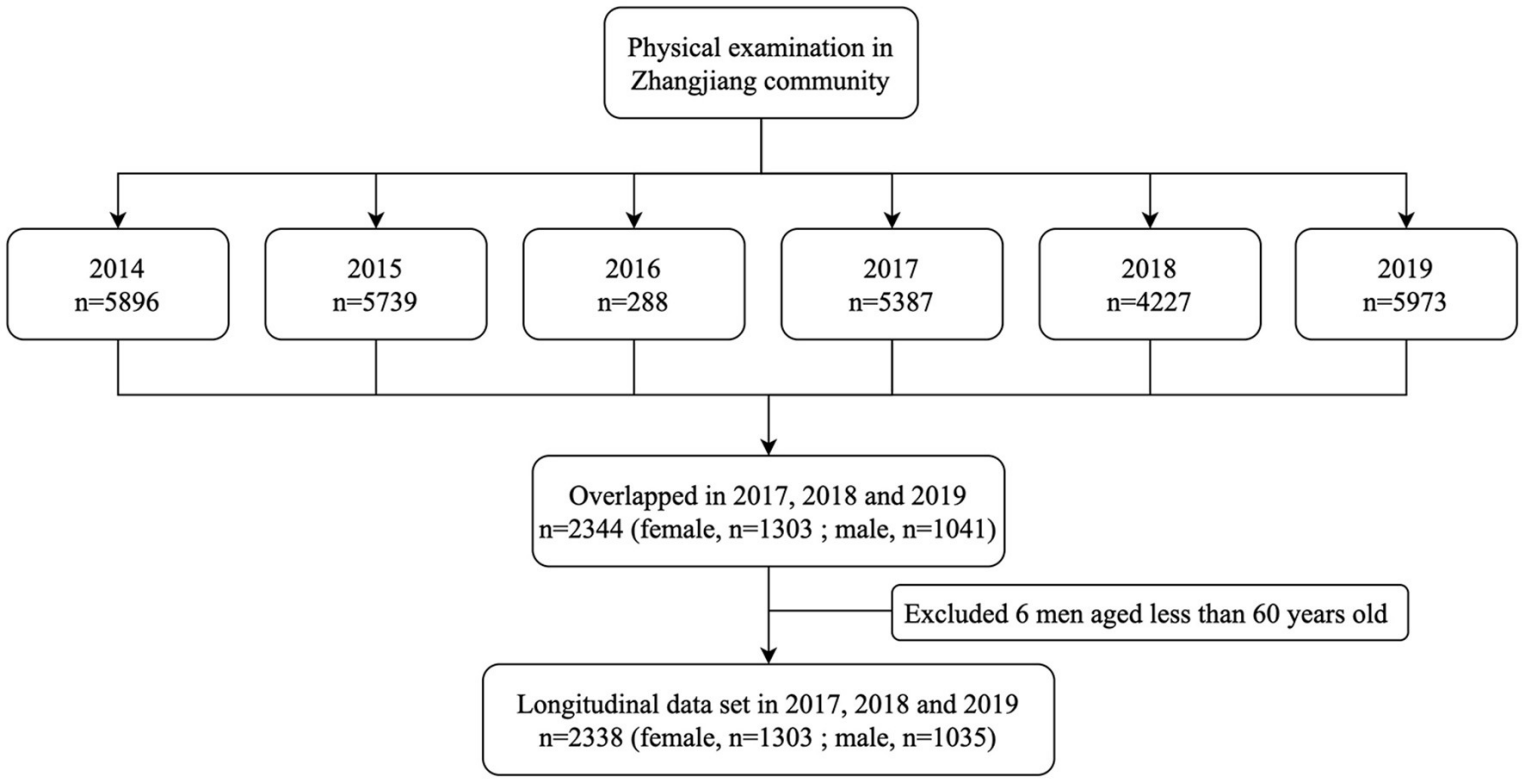
Study flowchart.

Participants were recruited from the outpatient registration pool of those who participated in annual health checks from 2014 to 2019 at the Zhangjiang area of Pudong District Health Care Service Centers, Shanghai, China. The study followed the Helsinki Declaration. A standard protocol has been developed by the Shanghai Innovation Center of Traditional Chinese Medicine Health Service and approved by the Shanghai University of Traditional Chinese Medicine Ethics Committee. Consent was obtained from all subjects. Participants with age over 60 years, who live in Shanghai, can complete measurements and informed consent were included in the inclusion criteria. This study excluded participants with mental disorders, malignant tumors, or incomplete medical records. During the investigation, six male subjects with age < 60 years were excluded, resulting in a total of 2,338 (female, *n* = 1,303; male, *n* = 1,035) Chinese elderly subjects with complete data overlapped in 2017, 2018, and 2019 (Figure 1). To elucidate the association between genetic variants and NAFLD in the elderly Chinese Han population, we did a sub-analysis of SNPs in 2017.

### The questionnaire, anthropometry, and physical examinations

Collection of information such as age, gender, alcohol consumption, smoking and medical history were collected by questionnaire (Supplementary Table 1). Body mass index (BMI) was calculated as weight (kg) divided by height squared (m^2^). Electronic sphygmomanometers were used to measure blood pressure (Bio-space, Cheonan, South Korea). Blood pressure was measured by electronic sphyg-momanometers (Biospace, Cheonan, South Korea). Waist and hip circumference were reliably measured using a non-stretch tape by the trained professional. Blood samples from the antecubital vein after fasting overnight were collected in the morning. Fasting glucose, alanine transaminase (ALT), aspartate transaminase (AST), total cholesterol (TC), low-density lipoprotein (LDL), high-density lipoprotein (HDL), triglyceride (TG), hemoglobin, hemameba, erythrocyte, urea, uric acid, total bilirubin, creatinine and alpha-fetoprotein (AFP) were measured using the biochemistry analyzer (Hitachi, Tokyo, Japan). The tumor marker carcinoma embryonic antigen (CEA) was quantitatively determined by an electro-chemiluminescence immunoassay (ECLIA).

### Genotyping

Genomic DNA was extracted from venous blood leukocytes using the EZ1 DNA Blood 350 μL kit (Qiagen) according to the manufacturer's instructions for genotyping. Seven SNPs related to NAFLD relevant traits, including rs2071518 in cellular communication network factor 3 (*CCN3*), rs12409877 in leptin receptor (*LEPR*), rs10770141 in tyrosine hydroxylase (*TH*), rs4430796 in hepatocyte nuclear factor 1-beta (*HNF1B*), rs2292354 in G protein-coupled receptor kinase interactor 2 (*GIT2*), rs5186 in angiotensin II receptor type 1 (*AGTR1*) and rs2206277 in transcription factor AP-2 beta (*TFAP2B*) from NCBI database of SNP database (www.ncbi.nlm.nih.gov/SNP) were analyzed, and genotyped by matrix-assisted laser desorption/ionization time-off light mass spectrometer in MassARRAY Analyzer 4 platforms (Sequenom, San Diego, CA). Probes and primers were determined with online Assay Design Suite version 2.0 software. Polymerase chain reaction was performed according to the instructions of the manufacturers. More detailed information about primers and polymerase chain reaction conditions is available upon request.

### Statistical analysis

Shapiro-Wilk test was used to check the normality of the data using IBM SPSS Statistics (version 26.0). If data were not normally distributed, their natural logarithms were used. Clinical data were presented as mean and standard deviation. Categorical data were calculated as percentages. FLD index was calculated by the following formula: FLD index = BMI + TG + 3 × (ALT/AST ratio) + 2 × Hyperglycemia (presence of Hyperglycemia, 1; absence of Hyperglycemia, 0) (10). Hyperglycemia was defined as fasting glucose ≥6.1 mmol/L and/or 2-h glucose ≥7.8 mmol/L and/or a previous clinical diagnosis of type 2 diabetes (11). MS score was calculated by the following formula: MS score = 2^*^waist/height + fasting glucose/5.6 + TG/1.7 + SBP/130 - HDL/1.02 (male) or 1.28 (female) (12).

A hierarchical non-linear model with random effects was used to assess the temporal patterns of anthropometric and serum traits from 2017 to 2019 (MLwin 2.26 software, Multiple Project, Institute of Education, University of London, UK). Age was entered as the explanatory variable in the form of polynomial spline functions to explain the change of target variables over time. Additionally, to determine the longitudinal associations of the FLD index or MS score with serum traits, this hierarchical model analysis was also used with urea and TB as independent variables and FLD index or MS score as an outcome variable.

To assess the directional effect between MS score and TB in females or FLD index and urea in males from 2017 to 2019, a bivariate cross-lagged panel model (CLPM) was used to estimate the structural relations of repeatedly measured variables at three different time points. The auto-regressive part of the model indicates the temporal stability of the variables from one-time point to the next. Meanwhile, CLPM was used to assess reciprocal relationships between the variables at consecutive time points, that is, MS score and TB in females and FLD index and urea in males during the follow-up. Structural equation modeling was conducted by Lavaan in R software (13).

For the sub-analysis, allelic and genotypic distributions and Hardy-Weinberg equilibrium (HWE) were analyzed with the online software SHEsis (http://analysis.bio-x.cn/myAnalysis.php) (14). The association between each SNP with NAFLD in five genetic models (codominant, dominant, recessive, over-dominant and log-additive models, respectively) was analyzed by using “SNPassoc” R package (15). *P* < 0.05 was considered statistically significant.

## Results

### Changes in patterns of anthropometric and serum traits over the 3 years from 2017 to 2019

Characteristics of the study participants are shown in Table 1. The average ages of females and males were 71 (in 2017) and 73 years old (in 2019).

**Table 1:** Participants information of this longitudinal study.

	**Female (*****N*** = **1,303)**						**Male (*****N*** = **1,035)**					
	**2017**		**2018**		**2019**		**2017**		**2018**		**2019**	
	**Mean**	**SD**	**Mean**	**SD**	**Mean**	**SD**	**Mean**	**SD**	**Mean**	**SD**	**Mean**	**SD**
Age (years)	71	5.67	72	5.62	73	5.62	71	5.67	72	5.62	73	5.62
BMI (kg/m^2^)	24.20	3.49	24.66	3.46	24.91	3.49	24.32	3.19	24.87	3.29	25.03	3.21
SBP (mmHg)	143.70	21.74	142.27	21.26	143.57	19.85	142.29	21.24	140.48	19.96	140.42	18.69
DBP (mmHg)	81.87	11.22	75.86	9.45	77.33	7.41	80.72	11.54	76.37	9.27	77.84	7.85
Waistline (cm)	81.68	8.84	82.55	8.79	81.52	8.64	84.99	9.18	85.56	8.91	84.30	8.66
Albumin (g/L)	44.29	2.54	42.02	2.01	44.17	2.44	44.21	2.63	41.93	2.02	44.11	2.45
ALT (U/L)	22.97	13.79	19.89	12.18	21.26	26.67	24.58	13.48	21.74	14.68	22.28	13.12
AST (U/L)	23.73	8.74	22.39	9.96	22.49	16.92	23.32	7.85	22.24	11.55	21.64	7.17
Urea (mmol/L)	5.52	1.51	5.64	1.44	5.57	1.48	5.58	1.53	5.76	1.59	5.65	1.65
Fasting glucose (mmol/L)	6.09	1.52	6.05	1.68	6.07	1.71	6.20	1.64	6.15	1.71	6.11	1.69
Hemoglobin (g/L)	132.82	10.28	132.81	10.85	133.84	11.81	147.83	12.32	146.90	12.85	146.84	13.63
TC (mmol/L)	5.27	0.93	5.02	0.89	4.95	0.94	4.79	0.91	4.52	0.97	4.47	0.87
HDL (mmol/L)	1.30	0.28	1.30	0.28	1.32	0.30	1.18	0.25	1.17	0.24	1.19	0.27
LDL (mmol/L)	3.25	0.83	3.12	0.78	1.32	0.30	3.03	0.85	2.88	0.79	1.19	0.27
TG (mmol/L)	1.53	1.03	1.68	1.24	1.63	1.18	1.36	1.03	1.52	1.46	1.43	1.07
UA (μmol/L)	319.76	76.96	320.26	75.56	300.90	82.08	374.33	85.62	371.95	84.80	351.01	90.14
TB (μmol/L)	14.99	4.75	13.31	4.37	13.63	4.72	17.10	6.03	14.73	4.93	15.22	5.31
Creatinine (U/L)	62.79	14.42	61.97	14.69	66.53	17.47	79.01	17.64	78.78	18.69	83.43	22.86
FLD index	29.22	4.41	29.54	4.48	29.92	4.51	29.51	4.22	29.92	4.61	30.11	4.35
MS score	3.14	0.89	3.21	1.02	3.18	1.01	2.87	0.90	2.95	1.14	2.87	0.96

ALT, alanine aminotransferase; AST, aspartate aminotransferase; BMI, body mass index; DBP, diastolic blood pressure; FLD index, fatty liver disease index; HDL, high density lipoprotein; LDL, low density lipoprotein; MS score, metabolic syndrome score; SBP, systolic blood pressure; TB, total bilirubin; TC, total cholesterol; TG, triglyceride; UA, uric acid.

**Figure 2:**
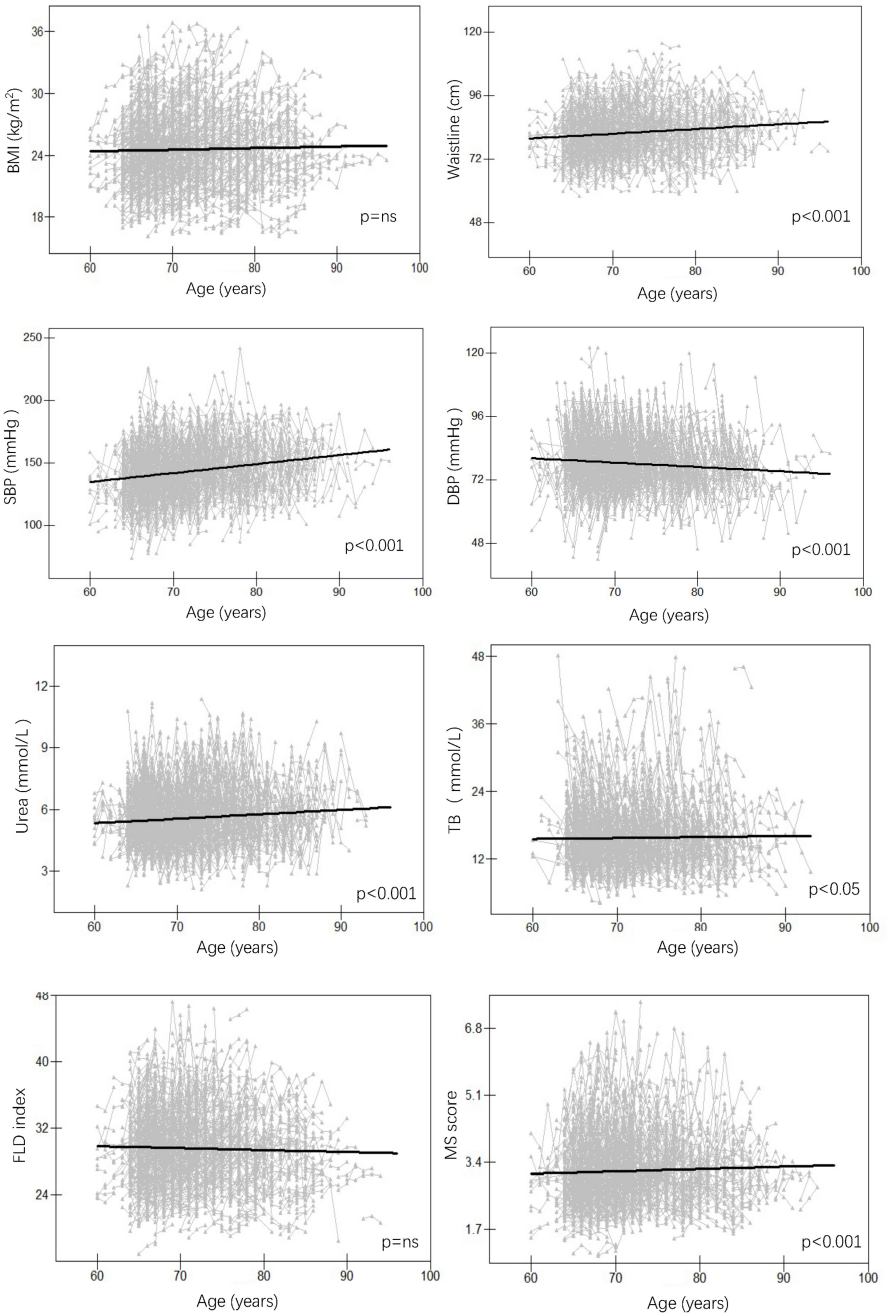
Longitudinal change pattern in females. BMI, body mass index; DBP, diastolic blood pressure; FLD index, fatty liver disease index; MS score, metabolic syndrome score; SBP, systolic blood pressure; TB, total bilirubin.

**Figure 3:**
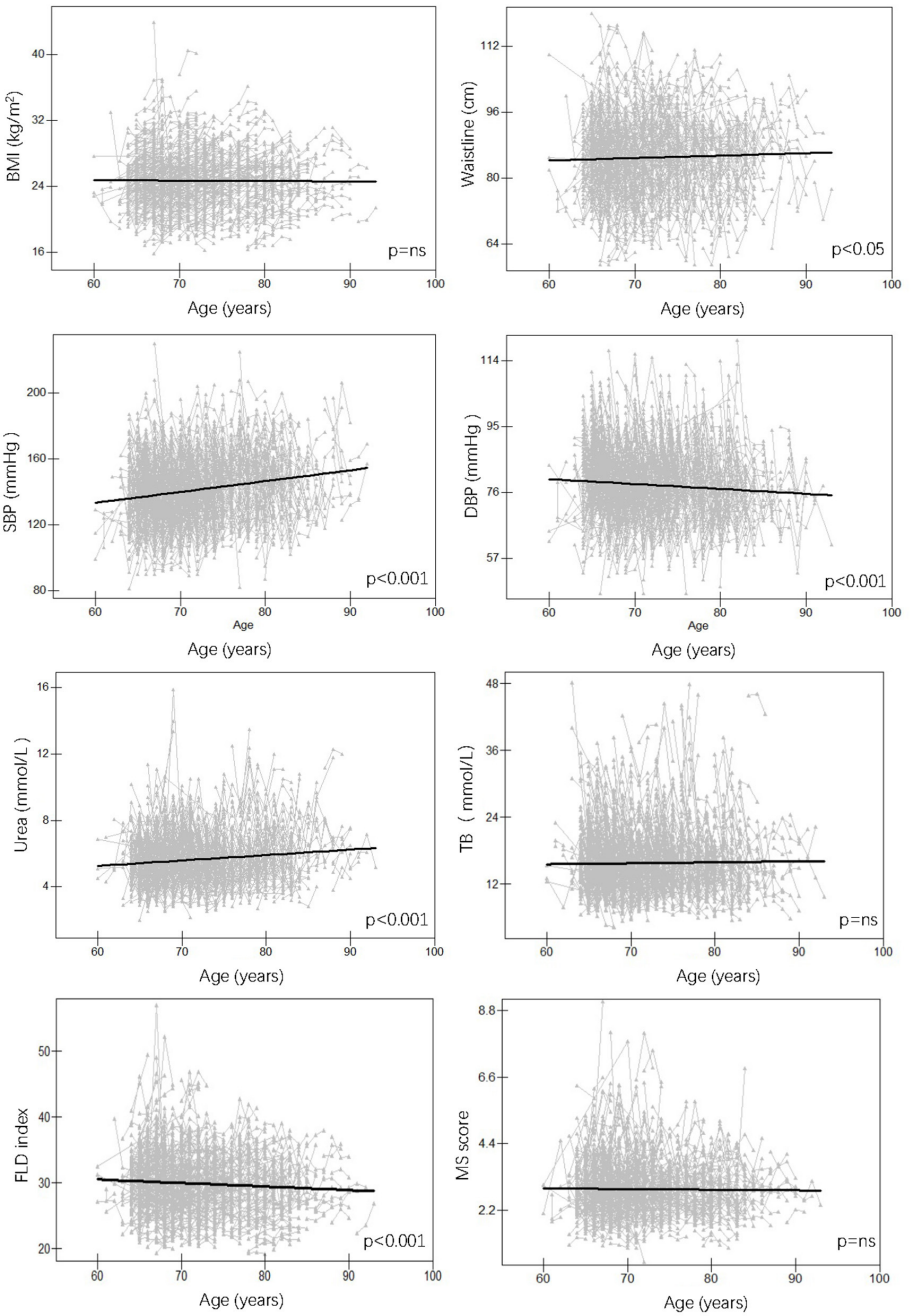
Longitudinal change pattern in males. BMI, body mass index; DBP, diastolic blood pressure; FLD index, fatty liver disease index; MS score, metabolic syndrome score; SBP, systolic blood pressure; TB, total bilirubin.

For anthropometric traits, waistline and SBP increased significantly over time, while DBP showed the opposite trend (*p* < 0.001 for all) and BMI showed no significant trend both in females (Figure 2) and males (Figure 3); For serum traits, albumin, erythrocyte, hemoglobin, ALT, TC, LDL and urea decreased steadily with aging, AST and HDL showed no significant trend in both females and males, while TB and glucose increased with aging only in females (*p* < 0.05 for all) (Supplementary Figures 1, 2). Notably, MS score increased with aging in females (*p* < 0.001), whereas the FLD index decreased with aging in males (*p* < 0.001).

### Longitudinal associations between MS score/FLD index and serum traits

**Figure 4:**
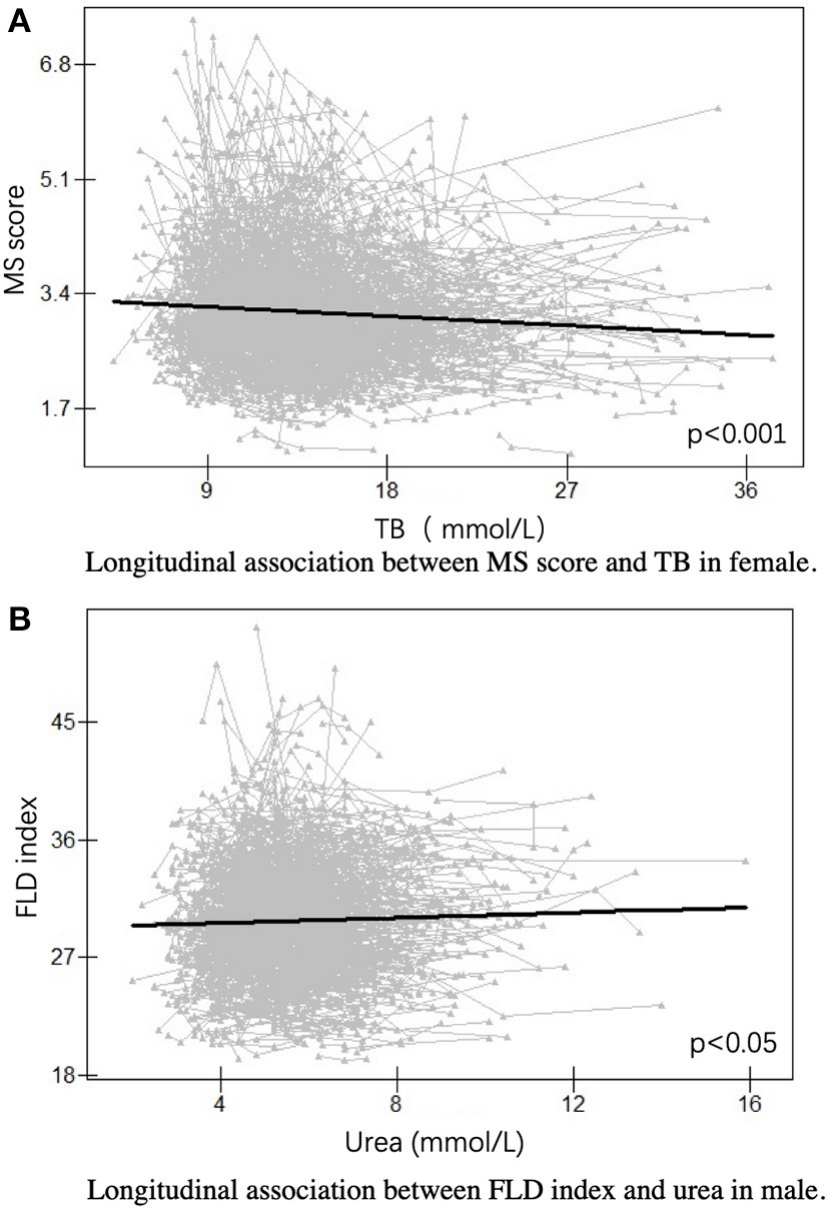
Longitudinal association in females and males. **(A)** Longitudinal association between MS score and TB in female. **(B)** Longitudinal association between FLD index and urea in male. FLD index, fatty liver disease index; MS score, metabolic syndrome score; TB, total bilirubin.

To further explore the mechanism differing in females and males, we assessed the longitudinal association between MS score or FLD index and serum traits in females and males. We found that MS score was negatively correlated with TB in females (*p* < 0.001), and FLD index was positively related to urea in males (*p* < 0.05) (Figure 4). Notably, no significant associations were found between MS score and urea in females or FLD index and TB in males.

### Bidirectional relationship between MS score/ FLD index and TB/urea in females/males

**Figure 5:**
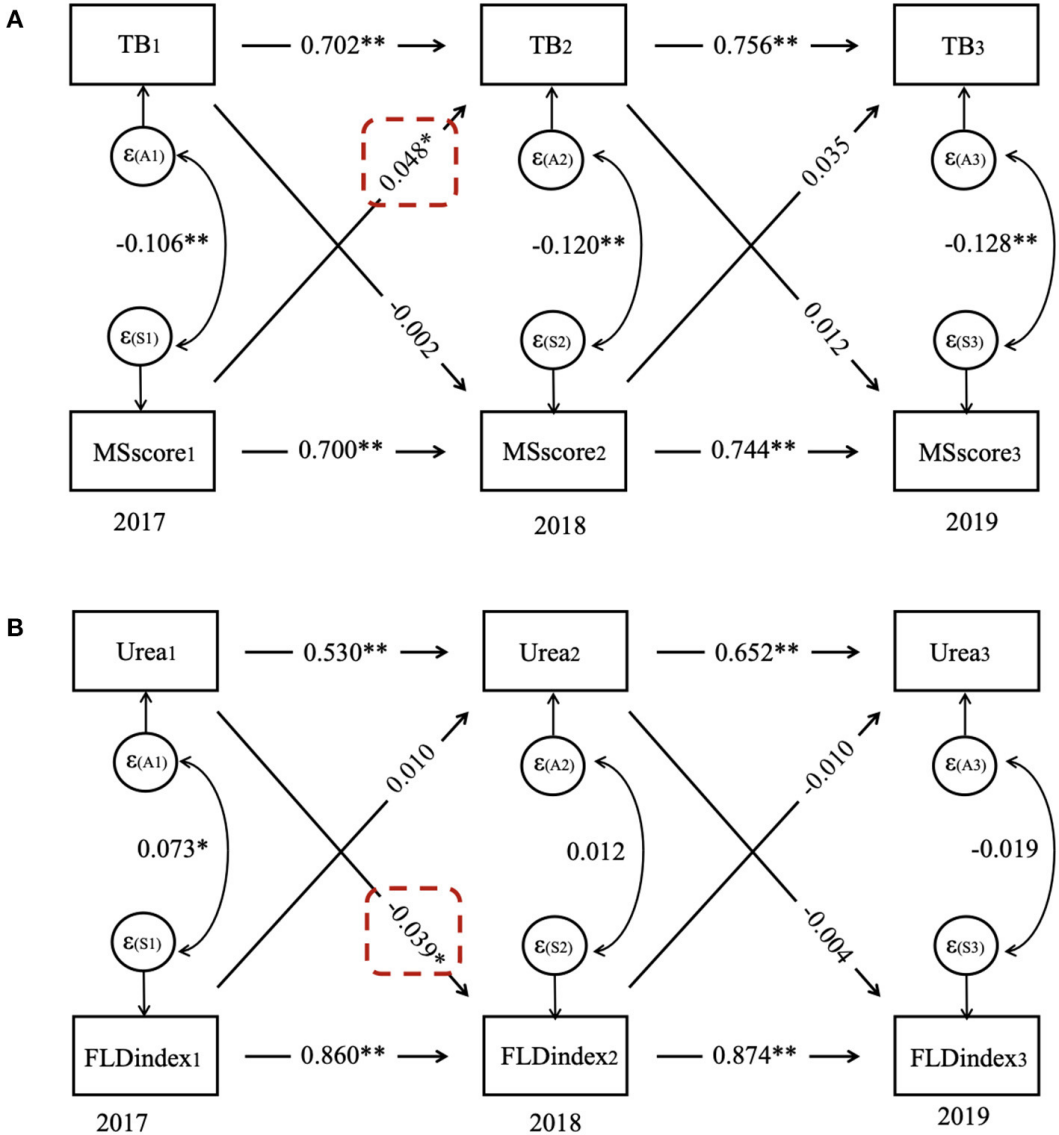
Cross-lagged panel model for **(A)** TB and MS score in females and **(B)** urea and FLD index in males. FLD index, fatty liver disease index; MS score, metabolic syndrome score; TB, total bilirubin.

In females, the CLPM showed that MS score predicted subsequent MS score at each time point (*p* < 0.001), and similar patterns were observed between the repeated measurements of TB (*p* < 0.001). The CLPM also showed that the MS score in 2017 predicted TB in 2018 (β = 0.048, *p* = 0.016), while TB in 2017 did not predict the MS score in 2018 (β = −0.002, *p* = 0.906) (Figure 5).

In males, the CLPM presented that the FLD index predicted subsequent FLD index at each time point (*p* < 0.001), the same as repeated measurements of urea (*p* < 0.001). Moreover, the CLPM also showed that urea in 2017 predicted the FLD index in 2018 (β = −0.039, *p* = 0.015), whereas the FLD index in 2017 did not predict urea in 2018 (β = 0.010, *p* = 0.718).

### Sub-analysis of the genetic variants in NAFLD

As genetic factors play essential roles in the occurrence and progression of NAFLD, we also conducted the SNPs test among 732 participants (NAFLD, *n* = 479; control, *n* = 253) in 2017 (Supplementary Table 2). There was no significant difference in age between NAFLD and control groups. The BMI of NAFLD patients was much higher than the controls (*p* < 0.001). The detailed information on seven SNPs is presented in Table 2.

**Table 2:** The SNPs analyzed in this sub-analysis.

**Gene**	**SNP ID**	**Chromosome**	**Function**	**Allele**
CCN3	rs2071518	8:119423572	3_prime_UTR_variant	T/C
LEPR	rs12409877	1:65478189	intron_variant genic_upstream_transcript_variant	A/G
TH	rs10770141	11:2172610	upstream_transcript_variant 5_prime_UTR_variant 2kb_upstream_variant	A/G
HNF1B	rs4430796	17:37738049	intron_variant	A/G
GIT2	rs2292354	12:109930396	3_prime_UTR_variant, genic_downstream_transcript_variant	G/A
AGTR1	rs5186	3:148742201	3_prime_UTR_variant	A/C
TFAP2B	rs2206277	6:50830813	intron_variant	C/T

AGTR1, angiotensin II receptor type 1; CCN3, cellular communication network factor 3; GIT2, G protein-coupled receptor kinase-interactor; HNF1B, hepatocyte nuclear factor 1-beta; kb, kilobase; LEPR, leptin receptor; SNPs, single nucleotide polymorphisms; TFAP2B, transcription factor AP-2 beta; TH, tyrosine hydroxylase; UTR, untranslated regions.

All the SNPs met HWE (*p* > 0.05). The allele frequencies of rs2071518, rs12409877, rs10770141, and rs4430796, and the genotype frequencies of rs2071518, rs10770141, rs2292354, rs5186, and rs2206277 were significantly different between NAFLD and controls (*p* < 0.05) (Table 3).

**Table 3:** Allele and genotype distribution in the subjects of the sub-analysis.

**SNPs**	**Allele frequency**		**X^2^**	**P**	**FDR**	**95% CI**	**Genotype frequency**			**X^2^**	**P**	**FDR**	**HWE**
rs2071518	T	C	6.876	0.008^**^	0.109	0.510–0.908	T/C	C/C	T/T	9.973	0.006^**^	0.085	0.700
NAFLD	132 (0.138)	824 (0.861)					98 (0.205)	363 (0.759)	17 (0.035)				
Non-NAFLD	96 (0.19)	408 (0.809)					78 (0.309)	165 (0.654)	9 (0.035)				
rs12409877	A	G	5.402	0.020^*^	0.241	0.419–0.931	A/A	G/G	A/G	5.263	0.071	0.419	0.539
NAFLD	866 (0.937)	58 (0.062)					407 (0.88)	3 (0.006)	52 (0.112)				
Non-NAFLD	448 (0.903)	48 (0.096)					203 (0.818)	3 (0.012)	42 (0.169)				
rs10770141	A	G	4.479	0.034^*^	0.343	0.428–0.97	A/G	G/G	A/A	8.82	0.012^*^	0.145	0.972
NAFLD	56 (0.06)	870 (0.939)					56 (0.120)	407 (0.879)	0 (0)				
Non-NAFLD	45 (0.09)	451 (0.909)					37 (0.149)	207 (0.834)	4 (0.016)				
rs4430796			4.107	0.042^*^	0.110	0.617–0.992	A/G	G/G	A/A	4.735	0.093	0.203	0.303
NAFLD	697 (0.733)	253 (0.266)					193 (0.406)	30 (0.063)	252 (0.53)				
Non-NAFLD	343 (0.683)	159 (0.316)					119 (0.474)	20 (0.079)	112 (0.446)				
rs2292354	G	A	2.225	0.135	0.637	0.627–1.065	G/A	G/G	A/A	7.84	0.019^*^	0.136	0.934
NAFLD	775 (0.810)	181 (0.189)					135 (0.282)	320 (0.669)	23 (0.048)				
Non-NAFLD	392 (0.777)	112 (0.222)					96 (0.380)	148 (0.587)	8 (0.031)				
rs5186	A	C	0.060	0.805	0.840	0.673–1.663	A/A	C/A	C/C	7.071	0.029^*^	0.437	0.991
NAFLD	888 (0.936)	60 (0.063)					414 (0.873)	60 (0.126)	0 (0)				
Non-NAFLD	470 (0.94)	30 (0.06)					223 (0.892)	24 (0.096)	3 (0.012)				
rs2206277	C	T	2.254	0.133	0.571	0.942–1.567	C/C	C/T	T/T	6.384	0.041^*^	0.41	0.229
NAFLD	707 (0.741)	247 (0.258)					273 (0.572)	161 (0.337)	43 (0.09)				
Non-NAFLD	393 (0.776)	113 (0.223)					150 (0.592)	93 (0.367)	10 (0.039)				

CI, confidence interval; FDR, false discovery rate; HWE, Hardy-Weinberg equilibrium; NAFLD, non-alcoholic fatty liver disease; SNPs, single nucleotide polymorphisms.

* and ** indicate p < 0.05 and p < 0.01, respectively.

As the longitudinal association between MS score and TB in females, FLD index and urea in males were different; here, we also checked the association of seven SNPs with NAFLD in females and males, respectively. In females, for rs12409877, A/G-G/G genotype under the dominant model (OR = 1.87, 95%CI = 1.07–3.26, *p* = 0.029) and A/G genotype under the overdominant model (OR = 1.90, 95%CI = 1.07–3.37, *p* = 0.031) were statistically related to increased risk of NAFLD, as well as the log-additive model (OR = 1.68, 95%CI = 1.03–2.76, *p* = 0.041), even after adjusting for age and BMI (FDR^1^ < 0.05); for rs10770141, A/G genotype under the codominant model (OR = 1.67, 95%CI = 0.94–2.96, *p* = 0.022) and A/G-A/A genotype under the dominant model (OR = 1.81, 95%CI = 1.03–3.17, *p* = 0.040) were significantly associated with increased risk of NAFLD, as well as the log-additive model (OR=1.89, 95%CI=1.11-3.22, p=0.022); for rs2292354, there was a significant association between G/A genotype under the overdominant model and the increased risk of NAFLD (OR = 1.54, 95%CI = 1.11–3.22, *p* = 0.022) (Table 4). Notably, we also analyzed the association between these SNPs, the MS score, and TB. We found that rs2292354 was significantly related to MS score in the dominant and overdominant genetic model (*p* = 0.025 and *p* = 0.023, respectively), as well as TB in the codominant and overdominant genetic model (*p* = 0.034 and *p* = 0.019, respectively) (Supplementary Table 3).

**Table 4:** Genotype distribution in five genetic models in females.

**Genotype**	**Genetic model**	**NAFLD (N)**	**Non-NAFLD (N)**	**OR**	**95% CI**		**p**	**FDR**	**p^1^**	**FDR^1^**
rs12409877	Codominant						0.089	0.111	0.034^*^	0.043^*^
	A/A	254	111	1.00						
	A/G	30	25	1.91	1.07	3.39				
	G/G	3	2	1.53	0.25	9.26				
	Dominant						0.029^*^	0.069	0.011^*^	0.028^*^
	A/A	254	111	1.00						
	A/G-G/G	33	27	1.87	1.07	3.26				
	Overdominant						0.031^*^	0.069	0.010^*^	0.028^*^
	A/A-G/G	257	113	1.00						
	A/G	30	25	1.90	1.07	3.37				
	log-Additive						0.041^*^	0.069	0.021^*^	0.036^*^
	0,1,2	287	138	1.68	1.03	2.76				
rs10770141	Codominant						0.022^*^	0.056	0.217	0.281
	G/G	255	111	1.00						
	A/G	33	24	1.67	0.94	2.96				
	A/A	0	2		0					
	Dominant						0.040^*^	0.067	0.225	0.281
	G/G	255	111	1.00						
	A/G-A/A	33	26	1.81	1.03	3.17				
	log-Additive						0.022^*^	0.056	0.167	0.281
	0,1,2	288	137	1.89	1.11	3.22				
rs2292354	Overdominant						0.049^*^	0.229	0.047^*^	0.202
	G/G-A/A	218	89	1.00						
	G/A	81	51	1.54	1.00	2.37				

CI, confidence interval; FDR, false discovery rate; NAFLD, non-alcoholic fatty liver disease; OR, odds ratio; SNPs, single nucleotide polymorphisms.

p^1^ and FDR^1^ indicate p value and adjusted p value by FDR method after adjusting age and BMI.

* and ** indicate p < 0.05 and p < 0.01, respectively.

In males, for rs2071518, T/C and T/T genotype under the codominant model (OR = 1.99, 95%CI = 1.17–3.39; OR = 1.17, 95%CI = 0.27–5.02, FDR = 0.049), T/C-T/T genotype under the dominant model (OR = 1.90, 95%CI = 1.14–3.17, FDR = 0.036) and T/C genotype under the overdominant model (OR = 1.98, 95%CI = 1.17–3.36, FDR = 0.036) were significantly related to increased risk of NAFLD, as well as the log-additive model (OR = 1.62, 95%CI = 1.03–2.53, FDR = 0.049), even after adjusting age and BMI (FDR^1^ < 0.05); for rs2206277, C/T and T/T genotype under the codominant model (OR = 0.85, 95%CI = 0.52-1.39; OR = 0.09, 95%CI = 0.01–0.71, FDR = 0.017) and T/T genotype under the recessive model (OR = 0.10, 95%CI = 0.01–0.75, FDR = 0.010) were significantly associated with the decreased risk of NAFLD, as well as the log-additive model (OR = 0.63, 95%CI = 0.42–0.95, FDR = 0.043), even after adjusting age and BMI (*p*^1^ < 0.05) (Table 5). No significant associations between above SNPs and FLD index or urea were found (Supplementary Table 3).

**Table 5:** Genotype distribution in five genetic models in males.

**Genotype**	**Genetic model**	**NAFLD (N)**	**Non-NAFLD (N)**	**OR**	**95% CI**		**p**	**FDR**	**p^1^**	**FDR^1^**
rs2071518	Codominant						0.039^*^	0.049^*^	0.027^*^	0.034^*^
	C/C	136	70	1.00						
	T/C	38	39	1.99	1.17	3.39				
	T/T	5	3	1.17	0.27	5.02				
	Dominant						0.015^*^	0.036^*^	0.008^**^	0.021^*^
	C/C	136	70	1.00						
	T/C-T/T	43	42	1.90	1.14	3.17				
	Overdominant						0.011^*^	0.036^*^	0.008^**^	0.022^*^
	C/C-T/T	141	73	1.00						
	T/C	38	39	1.98	1.17	3.36				
	log-Additive						0.035^*^	0.049^*^	0.019^*^	0.032^*^
	0,1,2	179	112	1.62	1.03	2.53				
rs2206277	Codominant						0.007^**^	0.017^*^	0.014^*^	0.036^*^
	C/C	97	71	1.00						
	C/T	66	41	0.85	0.52	1.39				
	T/T	15	1	0.09	0.01	0.71				
	Recessive						0.002^**^	0.010^*^	0.004^**^	0.020^*^
	C/C-C/T	163	112	1.00						
	T/T	15	1	0.10	0.01	0.75				
	log-Additive						0.026^*^	0.043^*^	0.048^*^	0.080
	0,1,2	178	113	0.63	0.42	0.95				

CI, confidence interval; FDR, false discovery rate; NAFLD, non-alcoholic fatty liver disease; OR, odds ratio; SNPs, single nucleotide polymorphisms.

p^1^ and FDR^1^ indicate p value and adjusted p value by FDR method after adjusting age and BMI.

* and ** indicate p < 0.05 and p < 0.01, respectively.

## Discussion

In the present study, we observed that the MS score increased with aging in females, and FLD index decreased with aging in males; further, the longitudinal negative association between MS score and TB in females, and positive association between FLD index and urea in males were found; additionally, it suggests potential causal associations between development in MS and increase of TB level in females, and rise in urea level and improved FLD index in males by CLPM. Additionally, this gender-specific distinction might be explained by the genetic variants.

Aging is accompanied with changes in body composition and blood traits. Waistline seems a better indicator to determine the health risk associated with obesity in the elderly (16). Lee et al. (17) reported that waistline was superior to BMI as a predictor of hypertension, dyslipidemia and type II diabetes in females and males. Our study also showed that waistline decreased with aging rather than BMI. Further, as cardiomyocyte has a limited capacity for regeneration and repair, evidence has shown that cardiac output decreases with aging (18). Instead, SBP increased with aging in our study, which was highly associated with arterial stiffness (19). In addition, decreased serum albumin, erythrocyte, hemoglobin, ALT, AST, and TC were strongly associated with aging and could reflect the inflammation, metabolic demand and several pathological conditions, including NAFLD, non-alcoholic steatohepatitis, fibrosis and hepatocyte carcinoma (20–27). Our results were consistent with the previous studies. Notably, TC and LDL levels declined with aging in both men and women, but HDL levels did not change much or even slightly increase with aging. This was supported by the finding from a cross-sectional study (28), and this phenomenon might suggest that people with longer life expectancy need maintain a high HDL concentration. All these physiological changes directly or indirectly led to the occurrence of MS and NAFLD with aging.

Aging seems to underlie many of the most prevalent chronic diseases, such as MS and NAFLD. In the present longitudinal study, we used MS score to quantify MS (12) as this continuous quantitative trait could reflect the changing pattern with aging and easily compare across studies and populations. TB was suggested as a biomarker to monitor the resistance against chronic diseases and successful aging (29). Kang et al. (30) found that individuals in the highest bilirubin quartile had a 41% reduced risk of coronary atherosclerosis compared with individuals in the lowest bilirubin quartile. Temme et al. (31) observed that the risk of cancer mortality decreased as bilirubin increased, and the effects were retained in the females but with no significance. Of note, Ong et al. (32) showed that the older females had higher TB levels, and the prevalence of self-reported cardiovascular diseases also tended to decrease with higher TB. These were in line with our results. Although TB increased with aging, MS score was negatively associated with TB in females. Besides, the MS score predicted TB level in females through CLPM. This finding might help answer the cause-and-effect relationship between MS and TB. Combined with the Kao' finding, which showing that estrogen receptor signaling could facilitate bilirubin metabolism (33), it may imply the importance of estrogen between TB and MS, especially for the elderly female population. This finding may also help us explain the link between TB and MS only occurring in the females. FLD index was also applied to reflect the extent of NAFLD (10) in our study. And FLD index declined with aging and urea changed in vice versa; urea predicted FLD index in males. Aging is the most common cause of NAFLD progression and is associated with changes in urea metabolism (9, 34). Our study provided more evidence on the causal link between NAFLD and urea.

For the above gender-specific pattern, it might be explained through genetic susceptibility. As genetic variants play essential roles in the occurrence and progression of chronic diseases, such as MS and NAFLD, here we found different associations between SNPs and the risk of NAFLD in females and males. And rs12409877 in *LEPR*, rs10770141 in *TH* and rs2292354 in *GIT2* were significantly associated with the increased risk of NAFLD in females; rs2071518 in *CNN3* and rs2206277 in *TFAP2B* were statistically related to the risk of NAFLD in males. Regarding the distinctive finding after gender stratification, we investigated SNPs' role in regulating NAFLD-related traits, such as TB and urea. It showed that rs2292354 in *GIT2* gene potentially regulated TB levels and MS in aging females. And this was supported by the following studies, *GIT2* was identified as a hub gene to connect with the aging process and aging-related diseases (35); as metabolic status influences aging, Martin et al. (36) deleted *GIT2* and found it altered transcriptomic signatures of the hypothalamus, which affects type II diabetes and metabolic pathways; *GIT2* is also highly responsive to oxidative stress (37); TB, as the end product of heme degradation, can improve the endothelial function of MS through inhibiting oxidative stress. All these clues suggested genetic variants in *GIT2* might play critical roles in the susceptibility of MS. Considering the possible sexual dimorphism, our results also highlighted the importance of consideration of gender in exploring risk factors for the progression in chronic diseases.

The main strength of this study is that longitudinal analysis was conducted in a homogenous, regionally representative cohort of aging females and males, and chronological patterns of anthropometric and blood traits were displayed. CLPM analysis was used to investigate the direction of associations between MS score and TB in females, and FLD index and urea in males, which allows for examining temporal associations better than logistic regression analysis and provides some clues to prevent the chronic diseases. Additionally, the associations of rs2292354 in *GIT2* with MS score and TB supported the gender-specific pattern in females in genetics. The limitation of this study is that the associations between MS score and TB in females, and FLD index and urea in males were required regardless of the effects of the hormone, while hormones might profoundly affect the chronic diseases in aging. In addition, since multiple factors would affect the gene variation, more research in different ethnic groups and regions with larger sample size are needed to verify the current result.

## Conclusions

Our study suggests the potential causal associations between development in MS and increase in TB level in females, and rise in urea level and improved FLD index in males. Moreover, the associations of rs2292354 in *GIT2* with MS score and TB in females were found. The SNP rs2292354 we investigated might be a biomarker for predicting MS in the elderly Chinese Han population.

## Data availability statement

The original contributions presented in the study are included in the article/Supplementary materials, further inquiries can be directed to the corresponding author/s.

## Ethics statement

The studies involving human participants were reviewed and approved by the Ethics Committee of Shanghai University of Traditional Chinese Medicine. All participants provided informed written consent prior to the study. The patients/participants provided their written informed consent to participate in this study.

## Author contributions

BL, GH, and YS designed research. NW, JL, NY, FZ, DL, and JW conducted research. NW and XZ analyzed and interpreted data. NW wrote the paper. BL, GJ, GH, LZ, and YS reviewed the manuscript critically. All authors have read and agreed to the published version of the manuscript.

## Funding

3-year action plan for Shanghai (project number: ZY (2021-2023)-0211), National Natural Science Foundation of China (81973730), Local Colleges Faculty Constitution of Shanghai MSTC 2022 (22010504300), Shanghai Collaborative Innovation Center for Chronic Disease Prevention and Health Services (2021 Science and Technology 02-37). Shanghai Health Commission for Traditional Chinese Medicine Research (2022QN014).

## Conflict of interest

The authors declare that the research was conducted in the absence of any commercial or financial relationships that could be construed as a potential conflict of interest.

## Publisher's note

All claims expressed in this article are solely those of the authors and do not necessarily represent those of their affiliated organizations, or those of the publisher, the editors and the reviewers. Any product that may be evaluated in this article, or claim that may be made by its manufacturer, is not guaranteed or endorsed by the publisher.
