# Supplementary material for: The gender-specific bidirectional relations between chronic diseases and total bilirubin/urea in the elderly population: A 3-year longitudinal study
Source: Front Public Health. 2022 Nov 9;10:1003505. doi: 10.3389/fpubh.2022.1003505 (PMC9682180; doi:10.3389/fpubh.2022.1003505)
Supplement: Supplementary file 1 [file Table_1.doc]

**Supplementary Table 1** Participants information of this longitudinal study

|  | Female | | | | | | | |  | Male | | | | | |
| --- | --- | --- | --- | --- | --- | --- | --- | --- | --- | --- | --- | --- | --- | --- | --- |
|  | 2017 | | 2018 | | | 2019 | | |  | 2017 | | 2018 | | 2019 | |
|  | Mean | SD | | Mean | SD | | Mean | SD |  | Mean | SD | Mean | SD | Mean | SD |
| Hemameba (10^12^/L) | 5.50 | 1.36 | | 5.43 | 1.27 | | 5.60 | 1.43 |  | 5.91 | 1.57 | 5.83 | 1.47 | 5.93 | 1.44 |
| Erythrocyte (10^12^/L) | 4.37 | 0.39 | | 4.42 | 0.42 | | 4.40 | 0.44 |  | 4.79 | 0.46 | 4.82 | 0.47 | 4.75 | 0.50 |
| AFP (ng/mL) | 6.80 | 4.91 | | 6.03 | 4.66 | | 4.98 | 4.01 |  | 6.81 | 5.78 | 6.00 | 4.04 | 5.36 | 7.49 |
| CEA (ng/mL) | 4.81 | 3.53 | | 4.77 | 4.70 | | 4.67 | 7.22 |  | 4.91 | 2.72 | 4.84 | 2.82 | 4.45 | 2.89 |
|  | N | % | | N | % | | N | % |  | N | % | N | % | N | % |
| NAFLD |  |  | |  |  | |  |  |  |  |  |  |  |  |  |
| Yes | 735 | 57.0 | | 967 | 74.8 | | 901 | 69.7 |  | 542 | 52.1 | 812 | 78.0 | 814 | 78.2 |
| No | 555 | 43.0 | | 325 | 25.2 | | 392 | 30.3 |  | 499 | 47.9 | 229 | 22.0 | 227 | 21.9 |
| MS |  |  | |  |  | |  |  |  |  |  |  |  |  |  |
| Yes | 432 | 33.5 | | 422 | 32.7 | | 414 | 32.0 |  | 170 | 16.3 | 170 | 16.3 | 159 | 15.3 |
| No | 858 | 66.5 | | 870 | 67.3 | | 879 | 68.0 |  | 871 | 83.7 | 871 | 83.7 | 882 | 84.7 |
| Osteoporosis |  |  | |  |  | |  |  |  |  |  |  |  |  |  |
| Yes | 9 | 0.70 | | 11 | 0.90 | |  |  |  | 4 | 0.40 | 5 | 0.50 |  |  |
| No | 1281 | 99.3 | | 1281 | 99.1 | | 1293 | 100.0 |  | 1037 | 99.6 | 1036 | 99.5 | 1041 | 100.0 |
| Cerebral infarction |  |  | |  |  | |  |  |  |  |  |  |  |  |  |
| Yes | 138 | 10.7 | | 173 | 13.4 | | 236 | 18.3 |  | 108 | 10.4 | 126 | 12.1 | 202 | 19.4 |
| No | 1152 | 89.3 | | 1119 | 86.6 | | 1057 | 81.7 |  | 933 | 89.6 | 915 | 87.9 | 839 | 80.6 |
| Current smoking |  |  | |  |  | |  |  |  |  |  |  |  |  |  |
| Yes | 4 | 0.31 | | 3.00 | 0.24 | | 3 | 0.24 |  | 84 | 8.07 | 76 | 7.3 | 78 | 7.49 |
| No | 1289 | 99.7 | | 1290 | 99.8 | | 1290 | 99.8 |  | 957 | 91.93 | 965 | 92.7 | 963 | 92.51 |
| Alcohol consumption |  |  | |  |  | |  |  |  |  |  |  |  |  |  |
| Yes | 61 | 4.72 | | 65 | 5.03 | | 68 | 5.26 |  | 235 | 22.3 | 213 | 20.5 | 202 | 19.4 |
| No | 1232 | 95.3 | | 1228 | 95.0 | | 1225 | 94.7 |  | 806 | 77.7 | 828 | 79.5 | 839 | 80.6 |

AFP, alpha fetoprotein; CEA, carcino-embryonic antigen; MS, metabolic syndrome; NAFLD, non-alcoholic fatty liver disease.

**Supplementary table 2** Participants information of the subanalysis

|  | NAFLD (N=479) | | Control (N=253) | | p |
| --- | --- | --- | --- | --- | --- |
|  | Mean | SD | Mean | SD |
| Age (years) | 72.14 | 5.14 | 72.40 | 5.27 | 0.882 |
| Female | 62.6% |  | 55.3% |  |  |
| Male | 37.4% |  | 44.7% |  |  |
| BMI (kg/m^2^) | 26.07 | 3.00 | 23.55 | 3.58 | <0.001** |

BMI, body mass index; NAFLD, non-alcoholic fatty liver disease. ** indicates p<0.01.

**Supplementary Table 3** Genetic association between SNPs and MS score/TB in females for the subanalysis

|  | | SNPs | Codominant | Dominant | Recessive | Overdominant | log-Additive |
| --- | --- | --- | --- | --- | --- | --- | --- |
|  | Females | | | | | | |
| With MS score | | rs10770141 | 0.416 | 0.965 | 0.195 | 0.760 | 0.840 |
|  | | rs12409877 | 0.577 | 0.829 | 0.295 | 0.911 | 0.630 |
|  | | rs2206277 | 0.896 | 0.744 | 0.665 | 0.932 | 0.664 |
|  | | rs5186 | 0.355 | 0.770 | 0.173 | 0.621 | 0.926 |
|  | | rs2292354 | 0.069 | 0.026* | 0.944 | 0.023* | 0.061 |
|  | | rs2071518 | 0.390 | 0.673 | 0.279 | 0.338 | 0.960 |
|  | | rs4430796 | 0.469 | 0.387 | 0.272 | 0.784 | 0.252 |
| With TB | | rs10770141 | 0.726 | 0.449 | 0.900 | 0.427 | 0.486 |
|  | | rs12409877 | 0.395 | 0.227 | 0.774 | 0.179 | 0.320 |
|  | | rs2206277 | 0.262 | 0.126 | 0.996 | 0.110 | 0.243 |
|  | | rs5186 | 0.270 | 0.462 | 0.128 | 0.604 | 0.352 |
|  | | rs2292354 | 0.034* | 0.106 | 0.144 | 0.019* | 0.417 |
|  | | rs2071518 | 0.284 | 0.787 | 0.118 | 0.651 | 0.430 |
|  | | rs4430796 | 0.493 | 0.895 | 0.278 | 0.462 | 0.720 |
|  | Males | | | | | | |
| With FLD index | | rs10770141 | 0.670 | 0.375 | 0.760 | 0.406 | 0.372 |
|  | | rs12409877 | 0.488 | 0.446 | 0.422 | 0.364 | 0.547 |
|  | | rs2206277 | 0.626 | 0.754 | 0.334 | 0.893 | 0.532 |
|  | | rs5186 | 0.311 | 0.734 | 0.170 | 0.474 | 0.988 |
|  | | rs2292354 | 0.600 | 0.476 | 0.610 | 0.349 | 0.660 |
|  | | rs2071518 | 0.820 | 0.535 | 0.786 | 0.590 | 0.532 |
|  | | rs4430796 | 0.578 | 0.363 | 0.760 | 0.296 | 0.512 |
| With urea | | rs10770141 | 0.686 | 0.394 | 0.727 | 0.433 | 0.385 |
|  | | rs12409877 | 0.936 | 0.987 | 0.721 | 0.938 | 0.965 |
|  | | rs2206277 | 0.369 | 0.191 | 0.380 | 0.355 | 0.157 |
|  | | rs5186 | 0.900 | 0.897 | 0.647 | 0.991 | 0.819 |
|  | | rs2292354 | 0.765 | 0.742 | 0.584 | 0.577 | 0.922 |
|  | | rs2071518 | 0.363 | 0.160 | 0.546 | 0.221 | 0.158 |
|  | | rs4430796 | 0.479 | 0.344 | 0.341 | 0.599 | 0.246 |

FLD, fatty liver disease; MS, metabolic syndrome; SNPs, single nucleotide polymorphisms; TB, total bilirubin.

**Supplementary Figure 1** Longitudinal change pattern in females


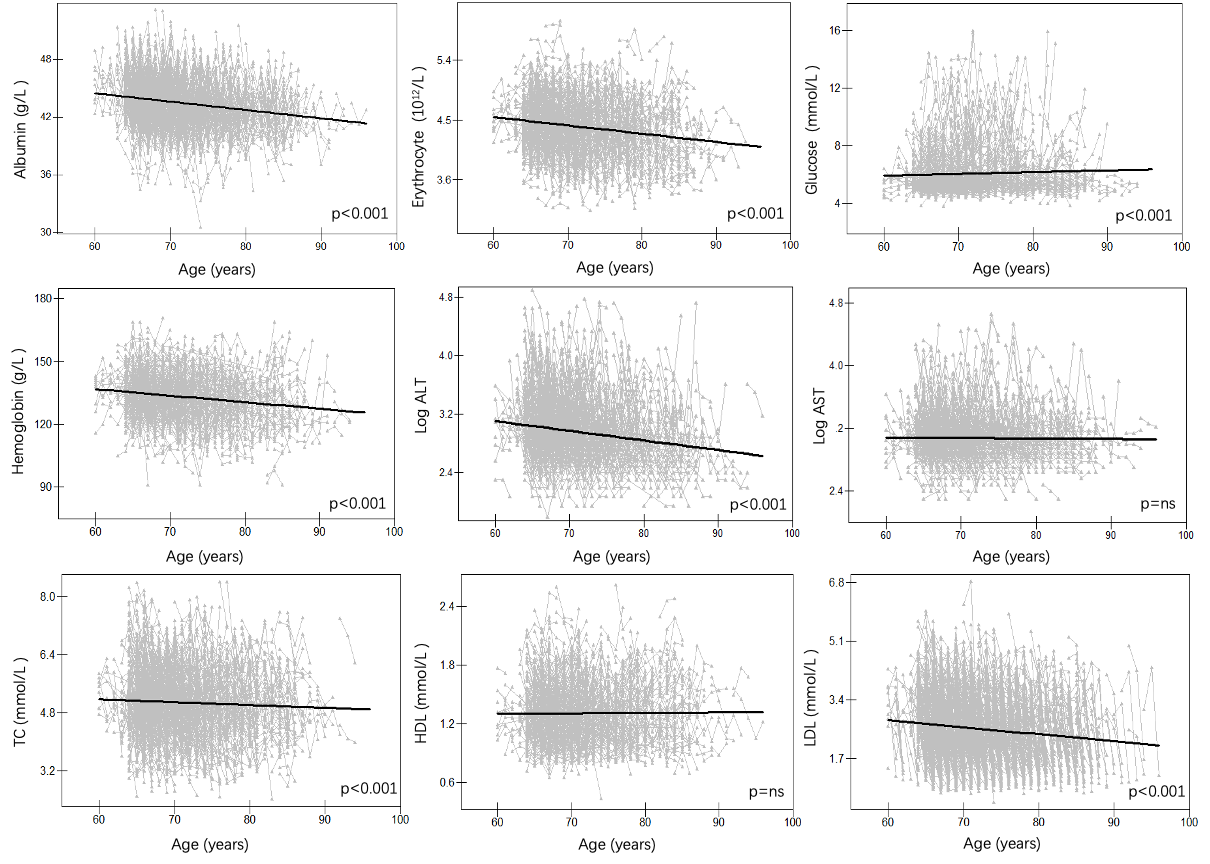


ALT, alanine aminotransferase; AST, aspartate aminotransferase; TC, total cholesterol; HDL, high density lipoprotein; LDL, low density lipoprotein.

**Supplementary Figure 2** Longitudinal change pattern in males

ALT, alanine aminotransferase; AST, aspartate aminotransferase; HDL, high density lipoprotein; LDL, low density lipoprotein; TC, total cholesterol.
